# Cloning, Expression, and Characterization of a Novel Thermophilic Monofunctional Catalase from* Geobacillus* sp. CHB1

**DOI:** 10.1155/2016/7535604

**Published:** 2016-08-07

**Authors:** Xianbo Jia, Jichen Chen, Chenqiang Lin, Xinjian Lin

**Affiliations:** Soil and Fertilizer Institute, Fujian Academy of Agricultural Sciences, Fuzhou 350003, China

## Abstract

Catalases are widely used in many scientific areas. A catalase gene (*Kat*) from* Geobacillus* sp. CHB1 encoding a monofunctional catalase was cloned and recombinant expressed in* Escherichia coli* (*E. coli*), which was the first time to clone and express this type of catalase of* genus Geobacillus* strains as far as we know. This* Kat* gene was 1,467 bp in length and encoded a catalase with 488 amino acid residuals, which is only 81% similar to the previously studied* Bacillus* sp. catalase in terms of amino acid sequence. Recombinant catalase was highly soluble in* E. coli* and made up 30% of the total* E. coli* protein. Fermentation broth of the recombinant* E. coli* showed a high catalase activity level up to 35,831 U/mL which was only lower than recombinant* Bacillus *sp. WSHDZ-01 among the reported catalase production strains. The purified recombinant catalase had a specific activity of 40,526 U/mg and *K*_*m*_ of 51.1 mM. The optimal reaction temperature of this recombinant enzyme was 60°C to 70°C, and it exhibited high activity over a wide range of reaction temperatures, ranging from 10°C to 90°C. The enzyme retained 94.7% of its residual activity after incubation at 60°C for 1 hour. High yield and excellent thermophilic properties are valuable features for this catalase in industrial applications.

## 1. Introduction

Catalases (EC1.11.1.6) are a class of enzymes that specifically catalyze the decomposition of H_2_O_2_ to H_2_O and O_2_ [1]. H_2_O_2_ has been applied to sterilization and bleaching processes in the medical, food, textile, and paper-making industries [2, 3]. Residual H_2_O_2_ in products or by-products is harmful to human health and environment. Thus, the removal of residual H_2_O_2_ is necessary in textile production processes, food health, pollution prevention, and other fields [2]. Catalase is an ideal choice for removing H_2_O_2_ due to its efficiency and lack of secondary pollution compared with chemical decomposition methods [4].

Catalase can be divided into four classes: monofunctional heme catalases, catalase-peroxidases, non-heme catalases, and minor catalases [5]. Catalases are widely present in animals, plants, fungi, most aerobic bacteria, and some anaerobic bacteria [5]. Commercial catalase produced by animals liver, plant tissues, and microbial fermentation is mainly mesophilic catalase, but many industrial applications need thermostable catalase; for example, textile bleaching temperature is always up to 60°C [6], so thermophilic catalase is more comparable on that condition. There have been some thermophilic catalases reported [7–10], but they were either catalase-peroxidase or Mn-dependent catalase. Thermophilic monofunctional heme catalases are rarely reported as we know.

High yielding strains are also necessary for enzyme production in fermentation processes. Recombinant expression is a practical method to increase the yield of a target gene. Currently,* Pichia pastoris *[11],* Hansenula polymorpha *[12],* Lactococcus lactis *[13],* Bacillus subtilis* (*B. subtilis*) [14, 15], and* Escherichia coli *(*E. coli*) [9] are used as host cells to produce recombinant catalase, but those recombinant catalases were mainly mesophilic, successfully recombinant expression precedents of thermophilic catalases which were few. The advantages of* E. coli* systems, such as their convenience, high yields, and ease of purification promote their wide application in genetic engineering. But many thermophilic enzymes cannot be overproduced in active forms in mesophilic host [8]. For example, though* Thermus thermophilus* catalase could solubly be expressed in* E. coli*, the recombinant catalase was absolutely inactive [8]; recombinant* E. coli* with* Bacillus stearothermophilus* catalase-peroxidase gene also showed a comparatively lower catalase level (1055.3 U/mL) [16]. So high soluble expression with an active form in* E. coli* is important for thermophilic catalase.

Previously, we screened thermophilic bacteria strains isolated from different high-temperature compost samples and* Geobacillus* sp. CHB1 was found to be a high catalase production strain. So, in this work, we cloned a novel* Kat* gene encoding a monofunctional catalase from thermophiles* Geobacillus* sp. CHB1 and recombinant expressed this gene in* E*.* coli* in a highly soluble form. The recombinant catalase was also purified and characterized.

## 2. Materials and Methods

### 2.1. Materials, Bacterial Strains, Plasmids, and Medium

*E. coli* BL21 (DE3) was cultured in our laboratory, and the expression vector pEASY-E2 was purchased from Beijing TransGen Biotech. The* Geobacillus* sp. CHB1 was isolated in Fuzhou, China. LB medium (10 g/L tryptone, 5 g/L yeast extract, 10 g/L NaCl, pH 7.0) was used in culturing* E. coli* BL21 (DE3). Auto-induction medium ZYM-5052 [17] was used for inducing the expression of recombinant catalase. The medium for culturing the* Geobacillus* sp. CHB1 consisted of soya peptone 0.9%, yeast extract 0.5%, NaCl 0.1%, K_2_HPO_4_ 0.1%, and KH_2_PO_4_ 0.075%, at pH 7.2.

### 2.2. Expression Vector Construction

*Geobacillus* sp. CHB1 was incubated at 60°C for 18 h at 180 r/min; then the genome was extracted according to the method of Zhou et al. [18]. The* Kat *gene was amplified using primers CatalaseF: (5′-GCAGATACAAAAAAGCTCACAAC-3′) and CatalaseR: (5′-TGCGTTTGTAATCACATCGTCCG-3′). Polymerase chain reaction (PCR) was performed with ExTaq DNA polymerase (TaKaRa, Dalian, China) under the following conditions: 95°C initial denaturation for 5 min, followed by 32 cycles of 40 s at 94°C, 40 s at 55°C, and 1 min 30 s at 72°C. The PCR product was purified using a PCR purification kit (Omega Bio-Tek, Inc., USA) and sequenced by Sangon Biotech Co., Ltd. (Shanghai, China).

Homology search of gene and amino acid sequence was carried out at BLAST server (http://blast.ncbi.nlm.nih.gov). Program blastp was used to analyze homology of the amino acid sequences; nonredundant protein sequences (Nr) database and Swiss Prot database were both used for blastp program. Sequences with high similarity in Swiss Prot database were selected to construct phylogenetic tree and do multiple alignment. MEGA 4.0.2 was used to construct the phylogenetic tree. Multiple alignment of the amino acid sequences was carried out using DNAMAN V6.0.3.99.

The purified fragment was ligated with pEASY-E2 using the described protocol of the kit. The ligated product was transformed into the competent* E. coli* cell strain Trans-T1 (TransGen Biotech, Beijing, China). A positive clone was selected using the T7 promoter primer (5′-TAATACGACTCACTATAGGG-3′) and CatalaseR (5′-TGCGTTTGTAATCACATCGTCCG-3′) and then cultured with LB medium containing 100 *μ*g/mL of ampicillin. The recombinant vector pEASY-E2-*Kat *was extracted using a Plasmid Mini Kit (Omega Bio-Tek, Inc., USA) and then transformed into competent* E. coli* BL21 (DE3) cells. A positive clone for BL21/pEASY-E2-*Kat* was selected using PCR as described above.

### 2.3. Inducing Expression and Purification of Recombinant Catalase

Positive clones were cultured in 5 mL of LB medium containing 100 *μ*g/mL of ampicillin at 37°C at 220 rpm for 12 h. Next, 1 mL of the above culture was inoculated into 50 mL of ZYM-5052 medium and was cultured at 30°C and 220 rpm for 16 h to induce the expression of the enzyme. To confirm expression, 8 mL of the induced culture was centrifuged at 10,000 ×g for 2 min and then suspended with 3 mL PBS (50 mM, pH 7.0). The suspended culture was subjected to ultrasonication at 400 W until clear. Then, 200 *μ*L of ultrasonicated sample was centrifuged at 12,000 ×g for 5 min at 4°C, and the supernatant was transferred to a new centrifuge tube. The supernatant and sediment sample were both subjected to 12% SDS-PAGE to detect the expression of the recombinant enzyme; a sample containing an empty vector was used as a blank control. Electrophoresis parameters were set as follows: 30 V for 30 min, followed by 80 V until the end. The coomassie brilliant blue R250 protocol [23] was used to dye the gel.

### 2.4. Recombinant Strain Enzyme Production Curve

First, 1 mL of seed liquid was inoculated into shake flasks containing 50 mL of ZYM-5052 medium, and the flasks were placed in shaking tables at 220 rpm and 30°C. Every 4 h, samples were collected and analyzed for catalytic activity. The samples were ultrasonicated before activity measurement collection. Enzyme production curves were then obtained.

### 2.5. Purification of Recombinant Catalase

First, 100 mL of the induced recombinant* E. coli* was centrifuged at 10,000 ×g for 5 min and washed once with Buffer A (50 mM NaH_2_PO_4_, 300 mM NaCl, pH 8.0). After centrifugation, the recombinant* E. coli* pellet was resuspended with 3 mL of Buffer A. The resuspended sample was ultrasonicated until clear. The ultrasonicated sample was centrifuged, and the supernatant was subjected to a Nickel-iminodiacetic acid (Ni-IDA) column. Then, 10 volumes of Buffer A (50 mM NaH_2_PO_4_, 300 mM NaCl, pH 8.0) were used to wash proteins that were nonspecifically bound to the column. Then, 10 volumes of Buffer B (50 mM NaH_2_PO_4_, 300 mM NaCl, 20 mM imidazole, pH 8.0) were used to remove other proteins. Finally, 5 volumes of Buffer C (50 mM NaH_2_PO_4_, 300 mM NaCl, 100 mM imidazole, pH 8.0) and 5 volumes of Buffer D (50 mM NaH_2_PO_4_, 300 mM NaCl, 500 mM imidazole, pH 8.0) were applied to elute the recombinant catalase. The purity of catalase was confirmed by SDS-PAGE. The purified catalase was placed into a dialysis bag and dialyzed against Buffer A to remove the high concentrations of imidazole. Buffer A was replaced every several hours until the imidazole was removed. The quantity of purified catalase was assayed using the Bradford method [24].

### 2.6. Recombinant Catalase Characteristics

A catalase activity assay was performed according to the method described by Beers Jr. and Sizer [25]. Purified recombinant catalase was diluted several times with PBS (20 mM, pH 7.0) to a suitable concentration, and 2.7 mL of 30 mM H_2_O_2_ (diluted with 20 mM PBS, pH 7.0) was then immediately added to 300 *μ*L of diluted enzyme. The absorbance changes at 240 nm were measured every 5 s for 1 min. 1 U of activity was defined as the amount of enzyme to decompose 1 *μ*mol of H_2_O_2_ per min. Each measurement was performed at least three times.

To assess the optimal reaction temperature, 30 mM H_2_O_2_ was preincubated at different temperatures from 10°C to 90°C (in 10°C increment), and then activities at different temperatures were measured with above preincubated H_2_O_2_.

To assess enzyme thermostability, diluted enzyme was incubated at 40, 50, 60, 70, 80, and 90°C for 30 min and 1 hour. After the incubation time, the heated enzyme was immediately transferred onto ice. Residual activities were measured with method described above.

The optimum pH was assayed with 30 mM H_2_O_2_ prepared in the following 50 mM buffers: sodium citrate 50 mM (pH 4–6), sodium phosphate 50 mM (pH 6–8), and glycine-NaOH 50 mM (pH 9–11). Activities at different pH values were measured as above in least three replicates.

Kinetic parameters were assayed by calculating the activity of the enzyme under different concentrations of H_2_O_2_ ranging from 2.5 to 25 mM in 20 mM PBS (pH 7.0) at 30°C. *K*_*m*_ was obtained by double-reciprocal plots according to Lineweaver and Burk [26].

## 3. Results and Discussion

### 3.1. *Kat* Sequence

**Figure 1:**
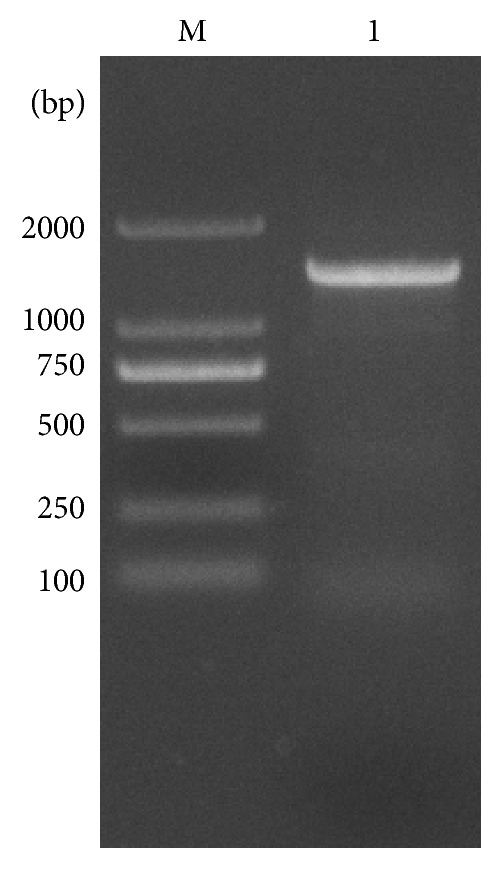
PCR product showing a 1,452-length band that corresponds to* Kat* from a* Geobacillus* sp. CHB1. Lanes: M, DNA marker; Lane 1, PCR product.

**Figure 2:**
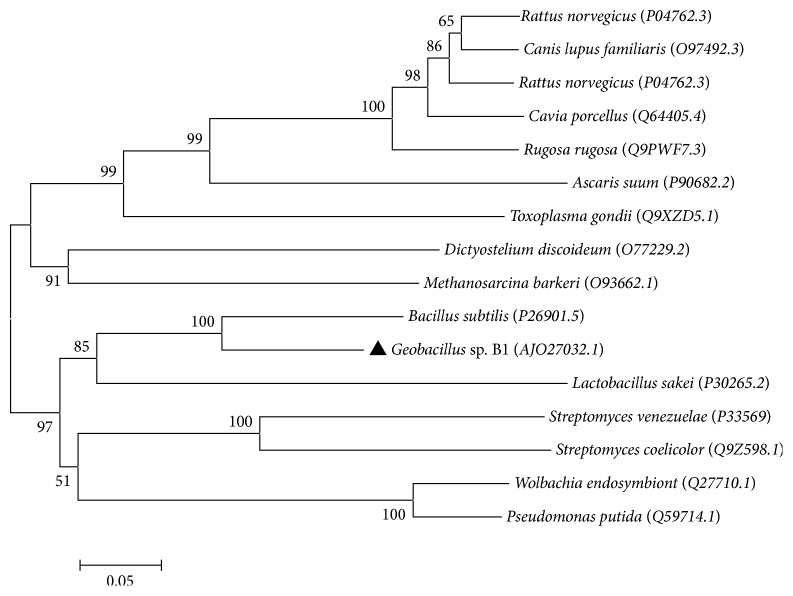
Phylogenetic tree of CHB1 catalase amino acid showing the relationship with other strains on catalase amino acid sequence. Protein sequences were selected by running blastp program with CHB1 catalase sequence in Swiss Prot database; accession numbers in brackets were the corresponding accession numbers of the strains in Swiss Prot database. Phylogenetic tree was constructed with MEGA 4.0.2 with the method Neighbor-Joining. Test of inferred phylogeny was Bootstrap for 1000 replications.

**Figure 3:**
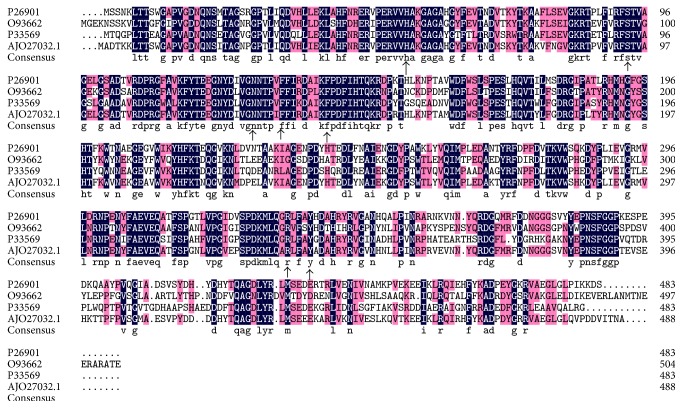
Multiple alignment of amino acid sequences for CHB1 catalase and catalase from Swiss Prot database. The sequences were those from* Bacillus subtilis* subsp. str. 168 (Swiss Prot number P26901),* Methanosarcina barkeri* str. Fusaro (Swiss Prot number O93662),* Streptomyces venezuelae* ATCC 10712 (Swiss Prot number P33569), and* Geobacillus* sp. CHB1 (GenBank number AJO27032.1). Identical amino acid residuals were shaded in black and conserved residuals were shaded in gray. Arrows showed the conserved residuals of home binding pocket of this kind of catalase.

The PCR product (Figure 1) and sequencing results showed that the total length of the* Kat *gene was 1,467 bp, which encoded a protein with 488 amino acids. Nucleotide sequence was submitted to GenBank, and GenBank accession number KP202252 was assigned. blastp was run in the nonredundant protein sequences of NCBI database for homologs of the amino acid sequence. The amino sequence had higher similarity with the catalase sequences of other* Geobacillus* spp.; however, all of these highly similar* Kat* genes were submitted in the form of sequenced genomes, and none had been previously cloned or expressed. Among all studied catalase sequences obtained from Swiss Prot database, this catalase was most similar to that of a* Bacillus* sp. [27], and its identity was 80%. Phylogenetic tree (Figure 2) based on the amino acid sequences similarity showed this catalase shared closest phylogenetic relationship with catalase of* Bacillus subtilis* and* Lactobacillus sakei *in Swiss Prot database. Multiple alignment of the amino acid sequence showed this catalase has seven typically conserved amino acid residuals for heme binding (Figure 3). All of the reported thermostable catalases [28, 29] from* Geobacillus stearothermophilus* were different from this catalase herein because they belonged to the second group of catalase family (catalase-peroxidase) and were encoded by* Per *gene, but catalase in this paper belonged to the first group (monofunctional heme catalase) which was encoded by gene* Kat *[5]. Therefore, to our knowledge this study was the first to clone and express this type of* Geobacillus* spp.* Kat* gene.

### 3.2. Construction of Recombinant Expression Vector pEASY-E2-*Kat*

**Figure 4:**
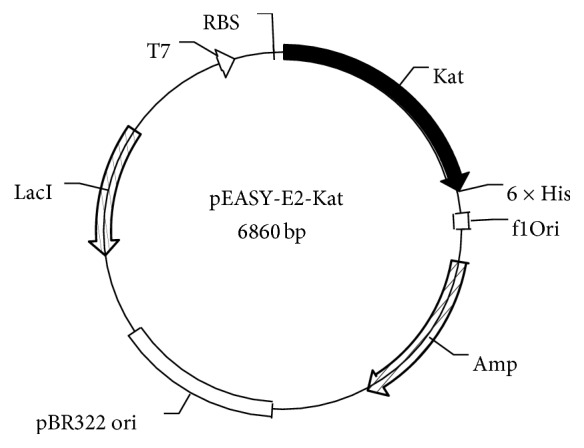
Recombinant expression construction of vector pEASY-E2-*kat*. A schematic diagram of recombinant expression vector pEASY-E2-*kat*. The* kat* gene encoding catalase was inserted into an expression vector with upstream RBS and T7 promoters and downstream 6×His.

**Figure 5:**
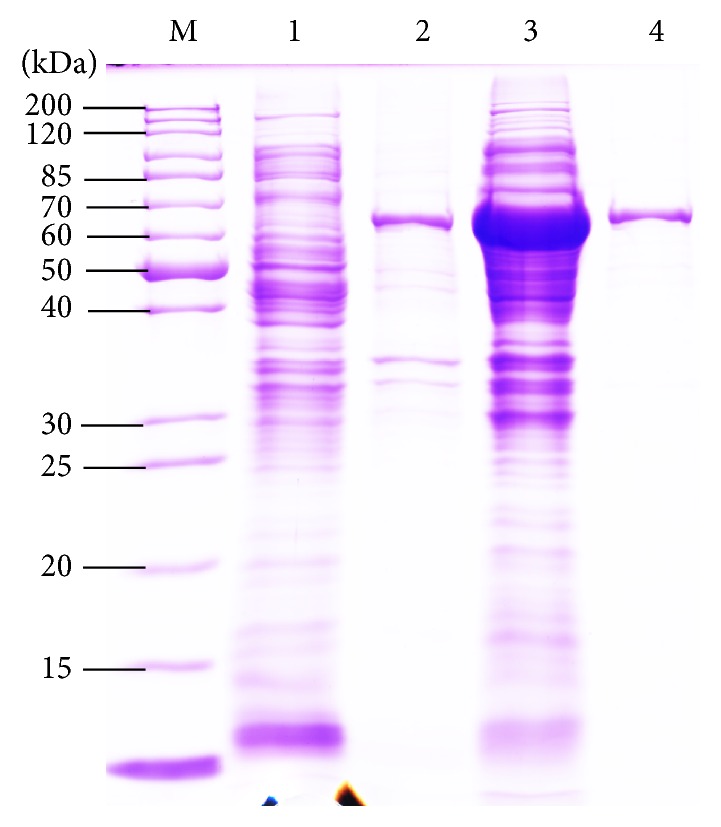
SDS-PAGE analyses of recombinant catalase expression and the purified enzyme. Lanes: M, protein marker; Lane 1, total bacterial protein of BL21 (DE3) cells with empty vector pEASY-E2; Lane 2, insoluble bacterial proteins of BL21 (DE3) with vector pEASY-E2-*kat* after induced expression; Lane 3, soluble bacterial proteins of BL21 (DE3) with vector pEASY-E2-*kat* after induced expression; Lane 4, SDS-PAGE analysis of purified catalase using a Ni-IDA column.

A positive clone was selected, and its sequence was confirmed by sequencing. The expression vector pEASY-E2-*Kat* (Figure 4) was transformed into BL21 (DE3) cells. Then, SDS-PAGE was applied to confirm whether the catalase was expressed and to determine the molecular weight of the enzyme. A band was observed between 60 and 70 kDa, and the recombinant catalase was highly soluble (make up to 30% of the total* E coli* protein) with a low inclusion body content (Figure 5). The theoretical molecular weight of the recombinant enzyme herein, calculated using its amino sequence, was 56 kDa, including the 6×His tag, which conflicted with the molecular weight of 65 kDa observed via SDS-PAGE. Some studies [30] have found that the 6×His tag might result in a recombinant protein with a greater molecular weight; however, the reason for this phenomenon remains unclear. The specific activity of the purified catalase was 40,526 U/mg under optimal conditions (60°C, pH 7.0).

### 3.3. Production Curve for Recombinant Catalase

**Figure 6:**
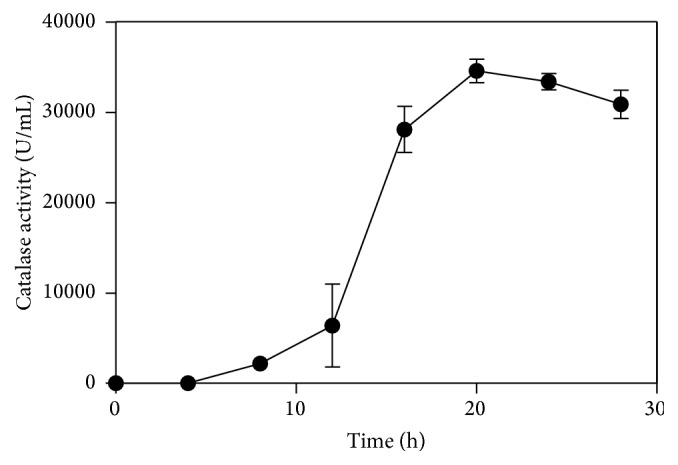
Catalase production curve of the recombinant* E. coli* BL21 (DE3).

Production curves for the recombinant catalase were constructed at 30°C. When cultured at 30°C for 20 h, the total activity of the fermentation broth reached a maximum level of 35,831 U/mL assayed under optimal conditions (Figure 6). This level was much higher than many catalase production strains and only lower than recombinant* Bacillus *sp. WSHDZ-01 (Table 1) as we know. High production ensured further application of this catalase.

### 3.4. Purified Recombinant Catalase Characteristics

#### 3.4.1. Effect of Temperature and pH on This Catalase

**Figure 7:**
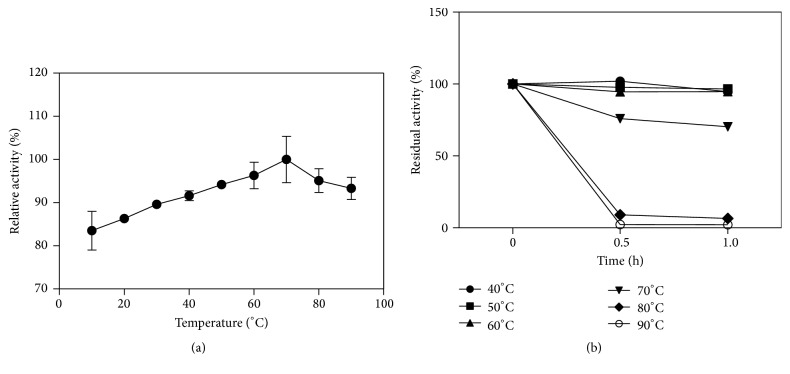
Effect of temperature on recombinant catalase activity. (a) Relative activity of the enzyme at 10, 20, 30, 40, 50, 60, 70, 80, and 90°C; (b) temperature stability of the enzyme. Residual activity of the recombinant enzyme by incubation at 40, 50, 60, 70, 80, and 90°C for 0.5 and 1 h.

**Figure 8:**
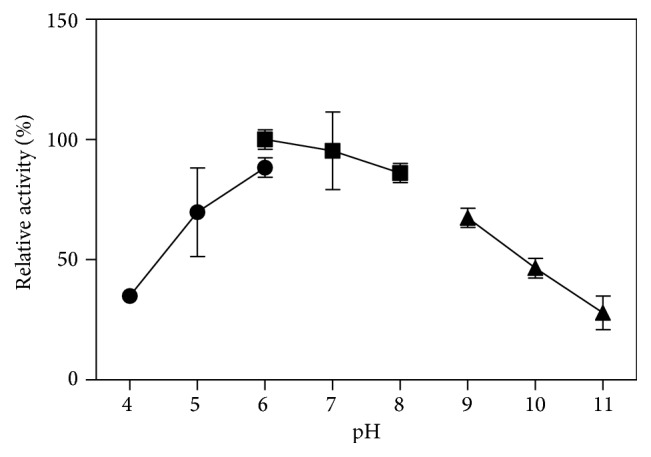
Optimal pH of the recombinant catalase. The buffers used for each pH region were 50 mM sodium citrate (pH 4–6), 50 mM sodium phosphate (pH 6–8), and 50 mM glycine-NaOH (pH 9–11). Each pH was assayed at least three times.

Temperature is a major factor in the application of many enzymes. The activity and stability of our purified catalase at different temperatures were assayed. The catalase in this study showed a high activity over a wide range of temperatures, from 10°C to 90°C (Figure 7(a)), and showed maximum activity at 60°C to 70°C. The temperature range was wider and optimal temperature was higher than many reported catalases, such as* Serratia marcescens* SYBC08 at temperatures ranging from 0°C to 70°C with optimal temperature of 20°C [31];* Psychrobacter piscatorii* T-3 from 10°C to 60°C with optimal temperature of 45°C [32]; and* Bacillus altitudinis* SYBC hb4 from 20°C to 40°C with optimal temperature of 30°C [33]. This catalase was stable at temperatures ≤60°C (Figure 7(b)) and when incubated at 70°C for 1 h, the enzyme maintained >70% residual activity; however, when the incubation temperature was >80°C, the residual activity was <10% after incubation for 1 h. Although this enzyme was inactive at ≥80°C, it still maintained high activity when added to reaction systems at 80°C and 90°C. Thermophilic catalase isolated from thermophilic* Thermoascus aurantiacus *[34] can retain 100% residual activity after incubation at 60°C for 1 h, which was similar to that of CHB1 catalase. This thermophilic property was much better than the properties of other catalases, such as those from* Psychrobacter piscatorii *(*P. piscatorii*) (65°C, 15 min, 20%) [32, 35],* Vibrio salmonicida *(60°C, 20 min, 0%) [36],* Vibrio rumoiensis* S-1T (65°C, 10 min, 0%) [37], and* Halomonas *sp. SK1 (55°C, 30 min, 0%) [38]. These properties enable the enzyme to be applied in both low and high-temperature processes in industry. The optimal pH of the enzyme was 6-7, and the enzyme retained >50% of its activity between pH 5 and 9 (Figure 8).

#### 3.4.2. Kinetic Parameters of This Catalase

**Figure 9:**
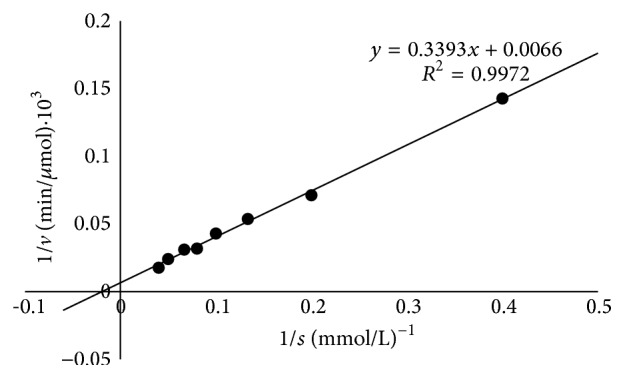
A Lineweaver–Burk plot of the recombinant catalase. Enzyme activity was assayed in 50 mM phosphate buffer (pH 7.0) and at 30°C.

The enzyme kinetics of the recombinant catalase were assayed using different H_2_O_2_ concentrations as substrates; a Lineweaver–Burk plot was used to calculate *K*_*m*_, 51.1 mM, and *V*_max_, 151.5 mol/min·mg (Figure 9), which is similar to previously reported values (52.5 mM [23] and 40.1 mM [2]). Many other catalases from* P. piscatorii *T-3,* Micrococcus luteus*,* Bacteroides fragilis*,* Helicobacter pylori*,* Serratia marcescens,* and* Xanthomonas campestris* have higher *K*_*m*_ values compared to the purified catalase [39]. The relatively low *K*_*m*_ indicates that the enzyme has a high affinity for H_2_O_2_ and that the enzyme is stable under high H_2_O_2_ concentrations. *K*_*m*_ is an important characteristic of enzymes and is vital for the assessment of their potential applications.

## 4. Conclusions

In this paper, we cloned, expressed, purified, and characterized a* Kat* gene of thermophilic bacteria* Geobacillus* sp. CHB1 for the first time. The recombinant enzyme could maintain its stability and showed a high activity over a wide range of temperatures from 10°C to 90°C. Specific activity of the purified recombinant catalase was 40,526 U/mg of protein, and recombinant* E. coli* BL21 strain reached a high level of catalase production to 35,831 U/mL. This enzyme's wide range of reaction temperatures, good thermostability, and high production may be suitable for the textile, paper-making, and other industries.

## Figures and Tables

**Table 1:** Catalase production levels of recombinant *E. coli* BL21 and other strains.

Strains	Activity (U/mL)	Reference
Recombinant* E. coli* BL21 (DE3)	35,831	This study
*Recombinant Bacillus *sp. WSHDZ-01	39,117	[15]
*Bacillus* sp. WSHDZ-01	28,990	[19]
*Exiguobacterium oxidotolerans* T-2-2T	22,000	[20]
*Serratia marcescens* SYBC08	20,353	[21]
*Rhizobium radiobacter* strain 2-1	17,035	[22]
*M. luteus*	6920	[22]
